# Clinical pathophysiology of hypoxic ischemic brain injury after cardiac arrest: a “two-hit” model

**DOI:** 10.1186/s13054-017-1670-9

**Published:** 2017-04-13

**Authors:** Mypinder S. Sekhon, Philip N. Ainslie, Donald E. Griesdale

**Affiliations:** 1 Division of Critical Care Medicine, Department of Medicine, Vancouver General Hospital, University of British Columbia, Room 2438, Jim Pattison Pavilion, 2nd Floor, 855 West 12th Avenue, Vancouver, BC V5Z 1M9 Canada; 2 Centre for Heart, Lung and Vascular Health, School of Health and Exercise Sciences, University of British Columbia Okanagan, Kelowna, BC Canada; 3 Department of Anaesthesiology, Pharmacology and Therapeutics, Vancouver General Hospital, University of British Columbia, West 12th Avenue, Vancouver, BC V5Z 1M9 Canada; 4 Centre for Clinical Epidemiology and Evaluation, Vancouver Coastal Health Research Institute, University of British Columbia, 899 West 12th Avenue, Vancouver, BC V5Z 1M9 Canada

**Keywords:** Hypoxic ischemic brain injury, Cardiac arrest, Cerebral oxygen delivery, Targeted temperature management, Cerebral edema, Carbon dioxide, Anemia, Hypothermia, Normobaric hyperoxia

## Abstract

Hypoxic ischemic brain injury (HIBI) after cardiac arrest (CA) is a leading cause of mortality and long-term neurologic disability in survivors. The pathophysiology of HIBI encompasses a heterogeneous cascade that culminates in secondary brain injury and neuronal cell death. This begins with primary injury to the brain caused by the immediate cessation of cerebral blood flow following CA. Thereafter, the secondary injury of HIBI takes place in the hours and days following the initial CA and reperfusion. Among factors that may be implicated in this secondary injury include reperfusion injury, microcirculatory dysfunction, impaired cerebral autoregulation, hypoxemia, hyperoxia, hyperthermia, fluctuations in arterial carbon dioxide, and concomitant anemia.

Clarifying the underlying pathophysiology of HIBI is imperative and has been the focus of considerable research to identify therapeutic targets. Most notably, targeted temperature management has been studied rigorously in preventing secondary injury after HIBI and is associated with improved outcome compared with hyperthermia. Recent advances point to important roles of anemia, carbon dioxide perturbations, hypoxemia, hyperoxia, and cerebral edema as contributing to secondary injury after HIBI and adverse outcomes. Furthermore, breakthroughs in the individualization of perfusion targets for patients with HIBI using cerebral autoregulation monitoring represent an attractive area of future work with therapeutic implications.

We provide an in-depth review of the pathophysiology of HIBI to critically evaluate current approaches for the early treatment of HIBI secondary to CA. Potential therapeutic targets and future research directions are summarized.

## Background

Cardiac arrest (CA) is a major cause of mortality and neurologic disability. The incidence of out-of-hospital CA is approximately 80 patients per 100,000 persons annually [1]. Despite advances in resuscitation, outcomes remain dismal, with 10% of patients surviving until hospital discharge and 5% experiencing full neurologic recovery [1].

The primary determinant of outcome after CA is hypoxic ischemic brain injury (HIBI). HIBI is the primary cause of death in 68% of inpatient CA and in 23% of out-of-hospital CA [2]. HIBI is associated with significant neurologic disability, ranging from mild cognitive deficits to minimally conscious and persistent vegetative states [2, 3]. Consequently, considerable effects on quality of life and incidence of psychiatric comorbidities, such as depression, anxiety, and posttraumatic stress disorder, are highly prevalent in HIBI survivors [4, 5]. The vast spectrum of acute and chronic HIBI phenotypes requires detailed understanding of cerebral physiologic perturbations that occur after CA and make clarifying the pathophysiology essential.

Management of HIBI is focused on limiting secondary injury [3] by optimizing the balance between cerebral oxygen delivery (CDO_2_) and use. Despite rigorous research, HIBI outcomes have not appreciably changed over 20 years [6, 7]. This stagnation is in contrast with improved outcomes in other critical care diseases [8].

Considerable opportunities remain to delineate the pathophysiology of HIBI. HIBI pathophysiology is a “two-hit” model, being determined by primary injury from immediate cessation of CDO_2_ during CA and secondary injury occurring after resuscitation. We present a narrative review of a two-hit model of HIBI pathophysiology as it pertains to physiologic parameters involved in maintaining the balance of CDO_2_ and use. We highlight advances pertaining to cerebral autoregulation, optimal hemoglobin, carbon dioxide, cerebral edema, normobaric hyperoxia, and targeted temperature management.

## Primary injury

**Fig. 1:**
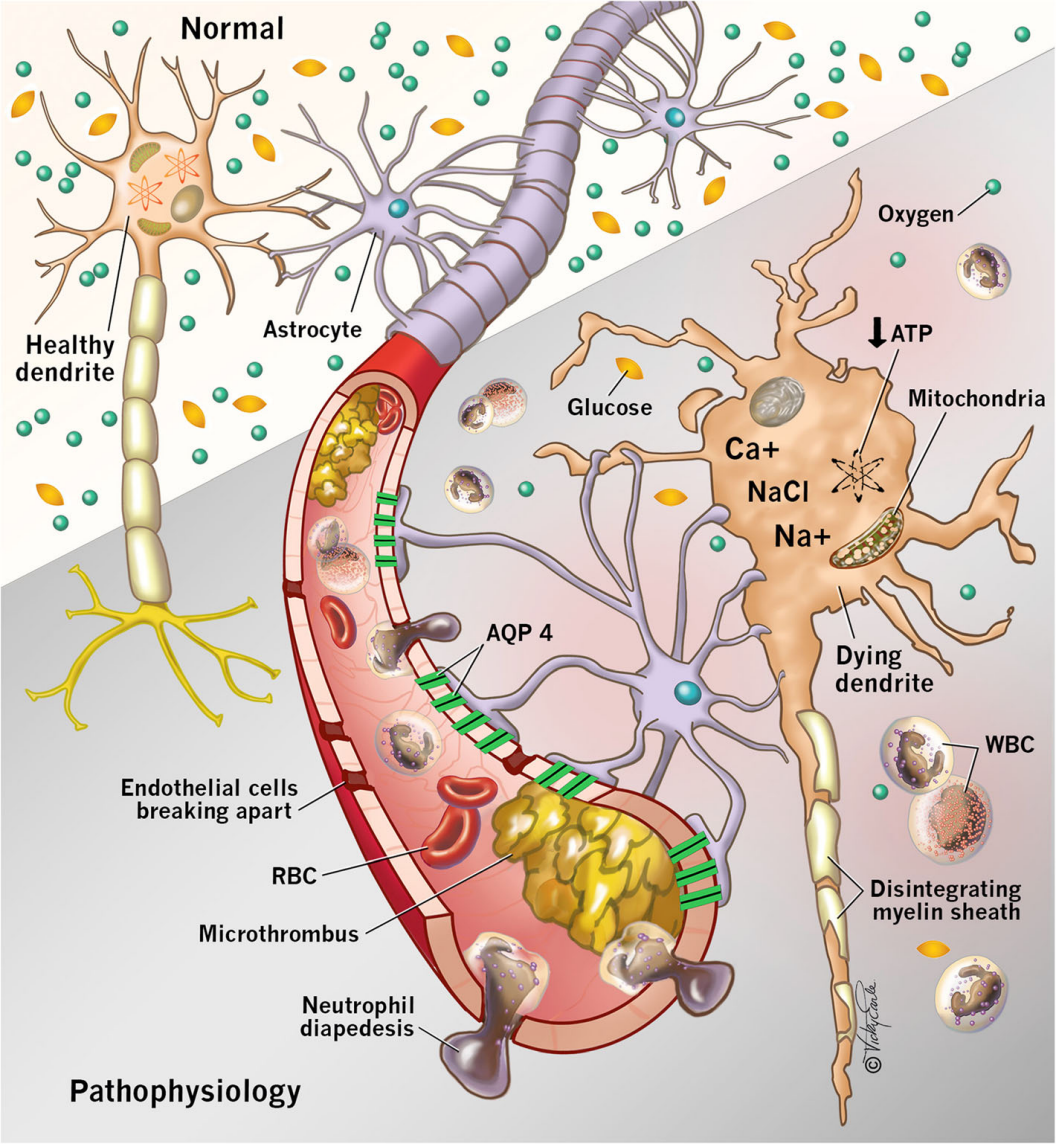
A schematic demonstrating the various microvascular and cellular pathophysiologic consequences which occur during the primary and secondary injury in hypoxic ischemic brain injury (HIBI). Decreased cerebral oxygen delivery manifests as reduced neuronal aerobic metabolism, causing reduced cellular adenosine triphosphate (ATP) production. Intracellular calcium accumulation leads to mitochondrial toxicity and further reduced ATP production. Inability to sustain cellular respiration results in cell death and apoptosis. Additionally, in the microvasculature, endothelial dysfunction leads to a porous blood-brain barrier, formation of cerebral edema, formation of microthrombi and limitation of cerebral blood flow with exacerbation of cellular ischemia. *AQP 4* Aquaporin-4, *RBC* Red blood cells, *WBC* White blood cells

During CA, cessation of CDO_2_ occurs with resultant neuron ischemia and cell death within minutes [9] (Fig. 1). The cerebrum consumes 20% to 25% of cardiac output to maintain function [3]. The brain is devoid of nutrient stores, and consequently neuroglycopenia and metabolic crisis occur within minutes after CA [10], leading to cell death.

As CDO_2_ decreases, adenosine triphosphate production halts, causing cessation of energy-dependent ion channel function [11]. Subsequent intracellular Na^+^ accumulation results in cytotoxic edema. Depletion of adenosine triphosphate leads to anaerobic metabolism, cerebral lactate accumulation, and intracellular acidosis [12]. Additionally, cellular ischemia causes intracellular Ca^2+^ influx through *N*-methyl-d-aspartate channels, which activates lytic enzymes [13] and mitochondrial dysfunction, thereby depleting adenosine triphosphate further [14]. Finally, excitatory neurotransmitter release activates lipases and proteases, which leads to apoptosis [15].

Clinically, loss of neurologic function is manifested by a decreased level of consciousness after global cerebral ischemia. Historically, Rossen et al. demonstrated that cessation of cerebral blood flow (CBF) by neck cuff insufflation to 600 mmHg in humans precipitated acute decreased level of consciousness within 10 seconds [16]. Decreased level of consciousness after CA occurs within 20 seconds after onset of ventricular fibrillation [17]. Loss of neurologic function has been demonstrated by isoelectric electroencephalography in observational studies [16]. Pana et al. identified human studies demonstrating isoelectric electroencephalography rhythms within 15 seconds and 30 seconds of asystole and ventricular fibrillation, respectively [16]. These findings are corroborated by animal studies establishing a similar timeline of 10 to 30 seconds from the onset of cerebral ischemia to isoelectric electroencephalography [18].

Although primary injury causes substantial neuronal loss, the ensuing postresuscitation additive cerebral injury accounts for significant cerebral ischemia and cellular death. The key pathophysiologic factors that are implicated in secondary injury are physiologic modifiers involved in maintaining the balance between CDO_2_ and use. We next discuss secondary injury and physiologic determinants that are targets of therapeutic interventions after HIBI.

## Secondary injury

**Fig. 2:**
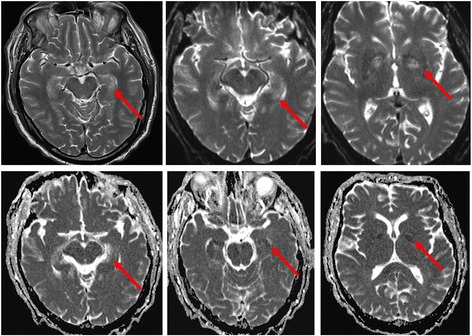
Magnetic resonance imaging sequences show focal hypoxic ischemic brain injury (HIBI) within the hippocampi and basal ganglia bilaterally. The images shown represent the acute changes after HIBI within the first week after resuscitation. In the top row, T2-weighted sequences reveal abnormal signaling in the hippocampi and basal ganglia as highlighted by the *red arrows*. In the bottom row, restricted diffusion-weighted imaging confirms HIBI in the affected regions of the hippocampi and basal ganglia as highlighted by the *red arrows*

Secondary injury is the additive cerebral injury characterized by an imbalance in postresuscitation CDO_2_ and use, ultimately culminating in neuronal death. It begins immediately after return of spontaneous circulation (ROSC). Structures especially susceptible include the hippocampi, thalami, cerebral cortex, corpus striatum, and cerebellar vermi [3] (Fig. 2), owing to highly metabolically active tissue. Aside from hypothermia, there are limited studies examining physiologic variables that exacerbate secondary injury. Table 1 summarizes the mechanisms of secondary injury.

**Table 1:** Summary of mechanisms of secondary brain injury after hypoxic ischemic brain injury

Pathophysiology	Mechanisms	Consequences
Microvascular dysfunction	Microthrombi, cerebral vasoconstriction, blood-brain barrier disruption	Increased cerebrovascular resistance, decreased CBF, decreased cerebral O_2_ delivery, vasogenic cerebral edema
Cerebral edema	Vasogenic cerebral edema, cytotoxic cerebral edema	Increased ICP and decreased CPP, decreased CBF, herniation, brain death
Anemia	Decreased arterial oxygen content	Cerebral ischemia
Impaired autoregulation	Narrowed and right-shifted autoregulation	Pressure passive cerebral hemodynamics, cerebral ischemia and hyperemia
Carbon dioxide	Hypocapnia-induced vasoconstriction, hypercapnia-induced vasodilation	Decreased CBF, cerebral ischemia, increased ICP, decreased CPP, decreased CBF
Hyperoxia	Increased O_2_ free radicals	Neuronal cell dysfunction and cell death
Hyperthermia	Increased CMRO_2_, decreased seizure threshold, induction of apoptosis	Neuronal cell metabolic crisis, cell death, nonconvulsive seizures, increased CMRO_2_, neuronal cell death

*Abbreviations*: *CBF* Cerebral blood flow, *ICP* Intracranial pressure, *CPP* Cerebral perfusion pressure, *CMRO*_*2*_ Cerebral metabolic rate of oxygen uptake

### Microcirculation and reperfusion injury

After ROSC, microcirculatory perturbations lead to further neuron dysfunction. The cerebrovascular endothelium plays a critical role in maintaining blood-brain barrier integrity, regulation of microcirculatory blood flow, and release of autoanticoagulant mediators [19]. Endothelial functions are compromised, and biomarkers of cerebrovascular endothelial injury are associated with adverse outcomes in HIBI [20].

Following ROSC, reperfusion injury causes neuronal dysfunction despite restoration of CDO_2_ [21]. An initial period of cerebral hyperemia is followed by hypoperfusion, resulting in a “no-reflow” [22] state that exacerbates secondary injury. Mechanisms implicated in the no-reflow state include impaired vasomotor regulation, decreased nitric oxide production, and resultant vasoconstriction [3, 19, 20]. Extravasation of intravascular water through a porous blood-brain barrier with perivascular edema leads to increased intravascular viscosity and cerebrovascular resistance [22]. Other mechanisms implicated in reperfusion injury include free radical release, glutamate production, and intracellular Ca^2+^ accumulation [23].

Endothelial autoanticoagulant dysfunction causes diffuse microthrombi in the cerebrovasculature [24]. Concomitant impaired vasodilation causes increased cerebrovascular resistance and reduces CBF [3, 22]. Interventional studies demonstrate that heparin and tissue plasminogen activator improve microcirculatory flow [25]. These findings have not translated into improved outcomes when evaluated prospectively, however [24, 26]. Finally, intravenous prostacyclin is suggested to promote endothelial function through vasodilatory and antiplatelet effects [19], but clinical studies are not yet available. Table 2 summarizes mechanisms involved in reperfusion injury.

**Table 2:** Pathophysiologic summary of cerebral reperfusion injury after cardiac arrest

Pathophysiology	Mechanisms	Consequences
Endothelial dysfunction	Impaired vasomotor control of blood flow, microthrombi formation, blood-brain barrier disruption	Impaired blood flow in microcirculation and limited oxygen delivery, cerebral edema
Free radical formation	Activation of lytic cellular enzymes	Neuronal apoptosis and cell death
Intracellular Ca^2+^ accumulation,	Mitochondrial toxicity, activation of cellular lytic enzymes	Reduced adenosine triphosphate production, cell death, apoptosis
Impaired nitric oxide,	Vasoconstriction, “no reflow”	Reduced cerebral blood flow, cerebral ischemia
Excitatory neurotransmitter release	Glutamate release	Excitotoxicity, seizures, apoptosis, cell death

### Hemoglobin

Hemoglobin is a major determinant of arterial oxygen content. In animal studies of traumatic brain injury, concomitant anemia exacerbates secondary injury from apoptosis [27]. However, physiologic benefits of improved CDO_2_ from transfusion must be balanced by risks associated with exogenous red blood cells. Although hemoglobin <70 g/L is the accepted transfusion threshold for nonbleeding critical care patients [28], it remains unclear if a liberal threshold is appropriate for patients with brain injury, who are susceptible to secondary injury from anemia [29].

Evidence of anemia in contributing to secondary injury in HIBI is limited to observational studies. Nakao et al. conducted a retrospective study of 137 subjects with witnessed CA and established that higher admission hemoglobin was an independent predictor of a 28-day favorable neurologic outcome (OR 1.26, 95% CI 1.00–1.58) [30]. These findings were corroborated by Wang et al., who demonstrated an association with adverse outcome and lower admission hemoglobin [31]. Recently, Johnson et al. conducted a multicenter observational study of 598 patients and found that favorable outcome patients had significantly higher hemoglobin (126 g/L versus 106 g/L, *p* < 0.001), a finding that persisted after adjustment [32].

Despite regression adjustment, admission anemia may be subject to strong residual or unmeasured confounding. It is unclear if admission hemoglobin captures the magnitude of effect that anemia has on secondary injury. Wormsbecker et al. accounted for this by investigating the relationship between mean hemoglobin over 7 days and neurologic outcome. They established that patients with a favorable outcome had significantly higher 7-day mean hemoglobin (115 g/L versus 107 g/L, *p* = 0.05) [33]. Furthermore, multivariable regression demonstrated that lower 7-day mean hemoglobin was associated with adverse outcome (OR 0.75 per 10 g/L change in hemoglobin, 95% CI 0.57–0.97) [33]. Importantly, Ameloot et al. established a link between hemoglobin and a measure of brain oxygenation in an observational study of 82 patients. They found a linear association between hemoglobin and brain regional saturation of oxygen (rSO_2_) using near-infrared spectroscopy [34], with hemoglobin <100 g/L being identified as a cutoff for lower rSO_2_ [34]. Additionally, they demonstrated that mean hemoglobin concentration <123 g/L was associated with worse neurologic outcome, particularly in patients with rSO_2_ < 62.5% (OR 2.88, 95% CI 1.02–8.16) [34]. Further research is required to establish an association between anemia with simultaneous brain hypoxia and investigate the effect of transfusion thresholds on outcome in HIBI.

### Carbon dioxide

Partial pressure of arterial carbon dioxide (PaCO_2_) modulates cerebrovascular resistance and CBF via its effects on vascular smooth muscle [35]. Specifically, hypocapnia (PaCO_2_ < 35 mmHg) induces cerebrovascular vasoconstriction and decreases CBF by about 2% to 3% for every 1 mmHg of PaCO_2_ [35]. Clinically, hypocapnia reduces intracranial pressure (ICP) by reducing cerebrovascular volume [35]. However, sustained hypocapnia can decrease CBF, increase cerebral oxygen extraction, and induce ischemia [36, 37]. Conversely, hypercapnia (PaCO_2_ > 45 mmHg) is a cerebrovascular vasodilator that causes hyperemia, exacerbates ICP [38], and reduces CBF [38]. Hypercapnia is also associated with excitotoxicity and increased cerebral oxygen demand [39]. Importantly, PaCO_2_ vascular reactivity is preserved after HIBI, making regulation of PaCO_2_ clinically significant and a crucial determinant of CDO_2_ [40]. The optimal PaCO_2_ in individual patients is not known but presents a unique opportunity for advanced neurophysiologic monitoring using transcranial Doppler ultrasonography to evaluate CBF, ICP, and cerebrovascular resistance with varying PaCO_2_ levels in HIBI.

Perturbations in PaCO_2_ in HIBI have been evaluated in observational studies of HIBI. Roberts et al. conducted a retrospective study of 193 patients and investigated the effects of hypocapnia and hypercapnia compared with normocapnia (PaCO_2_ 35–45 mmHg) on outcome. They demonstrated a relationship between adverse neurologic outcome and both hypocapnia (OR 2.43, 95% CI 1.04–5.65) and hypercapnia (OR 2.20, 95% CI 1.03–4.71) [35]. Exposure of hypocapnia and hypercapnia occurred 36% and 42% of the time after CA [35], respectively, making the exposure of CO_2_ fluctuation significant. The authors followed that study with an analysis of a prospective registry of patients with HIBI and found a significant association between normocapnia and good neurologic outcome (OR 4.44, 95% CI 1.33–14.85) [41]. Schneider et al. conducted a large multicenter database study of 16,542 patients with HIBI and investigated the effects of hypocapnia in HIBI, and they demonstrated a significant association between hospital mortality and hypocapnia (OR 1.12, 95% CI 1.00–1.24) compared with normocapnia [42]. Given the sound biological plausibility and available clinical data, regulation of PaCO_2_ warrants further systematic study to determine the precise optimal therapeutic strategy after HIBI. Critical links with intracranial physiologic parameters pertaining to ICP, CBF, and brain oxygenation and fluctuations in PaCO_2_ are logical future goals in this field.

### Cerebral edema

After HIBI, cerebral edema is a recognized complication that causes secondary injury. Because of a fixed overall intracranial volume, an increase in the parenchymal bulk from cerebral edema in HIBI can cause intracranial hypertension [43] with resultant decreases in cerebral perfusion pressure, CBF, and CDO_2_ [3]. This vicious cycle of cerebral edema precipitating increased ICP causes transtentorial herniation and brain death.

The origin of cerebral edema occurs as a result of either vasogenic or cytotoxic mechanisms. In the early stages, vasogenic edema emanates from fluid shifts from the intravascular to the cerebral interstitial space. Key to this process, aquaporin-4 is a membrane protein that transports water across cell membranes in the central nervous system. Aquaporin-4 proteins are located in perivascular astrocytic endfeet, processes, and ependyma [44]. The aquaporin-4 perivascular pool is identified as the predominant cluster involved in the pathophysiology of cerebral edema after HIBI, with increased aquaporin-4 expression occurring within 48 h after the onset of cerebral ischemia [44]. Interestingly, Nakayama et al. showed that 7.5% hypertonic saline attenuated cerebral edema in a wild-type mouse model of HIBI but had no effect in an aquaporin-4-knockout model, thereby demonstrating the importance of aquaporin-4 in the pathophysiology of cerebral edema and highlighting its therapeutic potential [44]. Hypertonic saline administration also restores blood-brain barrier integrity mediated by aquaporin-4 in the hippocampi, cerebellum, cortex, and basal ganglia [44]. Furthermore, Nakayama et al. established that achieving serum osmolality >350 mOsm/L with continuous infusion of conivaptan, a V_1_ and V_2_ antagonist, attenuated cerebral edema [45], thereby demonstrating that the effect of aquaporin-4 to decrease cerebral edema occurs through osmotic gradients, as opposed to a specific intravenous osmotic agent itself (e.g., 7.5% hypertonic saline).

Alternatively, cytotoxic edema originates from cellular metabolic crisis and intracellular energy depletion. Decreased adenosine triphosphate (Fig. 1) leads to energy-dependent ion channel failure and intracellular sodium and water retention. Rungta et al. established that the Na^+^Cl^−^ receptor SLC26A11 is a critical modulator of intracellular transport of chloride and subsequent cerebral edema after ischemia [46]. The authors showed that blockade of this receptor attenuated cytotoxic cerebral edema [46] after HIBI. The role of Na^+^Cl^−^ receptor antagonism after HIBI is yet to be clarified but represents a future therapeutic target.

Furthermore, sulfonylurea receptors are also implicated in the pathophysiology of cerebral edema after ischemia. Glyburide, a sulfonylurea receptor inhibitor, attenuates malignant cerebral edema after acute middle cerebral infarction [47]. These findings are corroborated by animal studies that demonstrate sulfonylurea receptor antagonism decreases cerebral edema after neuronal ischemia [48].

### Cerebral autoregulation

The brain has an innate ability to regulate blood flow to match metabolic demands. This phenomenon, termed *cerebral autoregulation*, allows the cerebrovasculature to undergo vasoconstriction and vasodilation over a range of mean arterial pressure (MAP) to maintain stable CBF [49]. Cerebral autoregulation mitigates the effects of hypoperfusion (ischemia) and hyperperfusion [49].

The identification of individualized MAP targets after HIBI using cerebral autoregulation monitoring is an attractive concept that has garnered significant interest. Initially, Nishizawa et al. demonstrated a linear relationship between MAP and CBF (as indexed by jugular venous oximetry) [50], suggesting complete dysfunctional cerebral autoregulation after HIBI. Thereafter, Sundgreen et al. constructed cerebral autoregulation curves for patients with HIBI by performing stepwise increases in MAP with norepinephrine and simultaneously estimating CBF with middle cerebral artery velocity on the basis of transcranial Doppler ultrasonography [51]. Of the 18 patients studied by Sundgreen et al., cerebral autoregulation was absent in 8 and present in 10 patients. In five of ten patients with preserved cerebral autoregulation, the lower limit of autoregulation was right-shifted with a median MAP 114 mmHg (range 80–120 mmHg) [51]. This sentinel study demonstrated the heterogeneous nature of cerebral autoregulation in patients with HIBI and suggested that the lower limit of autoregulation may be significantly higher than traditional MAP targets after HIBI.

**Fig. 3:**
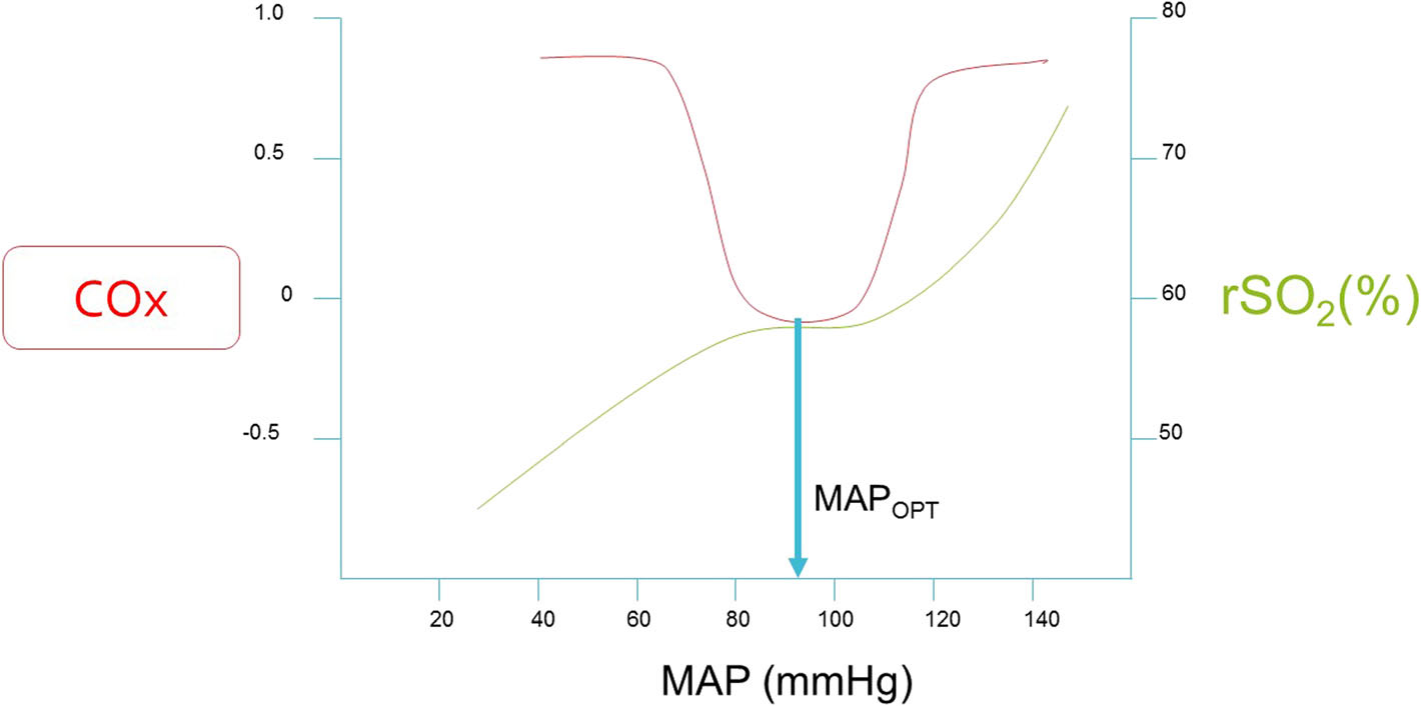
The zone of preserved autoregulation after hypoxic ischemic brain injury appears to be narrowed and right-shifted after cardiac arrest. Within the zone of autoregulation, regional saturation of oxygen (rSO_2_) is stable owing to the innate vasoconstriction and vasodilation of the cerebral vasculature to maintain stable cerebral blood flow. Outside the zone of autoregulation, a linear relationship exists between rSO_2_ and mean arterial pressure (MAP). By continually integrating the fluctuations of MAP and rSO_2_ with one another, a correlation coefficient (COx) can be generated. The COx approaches negative values or near-zero within the preserved zone of autoregulation, resulting in a U-shaped curve. The nadir of the U-shaped curve represents the optimal MAP (MAP_OPT_) for each individual patient

Recently, monitoring with near-infrared spectroscopy has garnered significant interest as a noninvasive method of optimal MAP identification and assessment of cerebral autoregulation after HIBI. Near-infrared spectroscopy measures the rSO_2_ in the outermost 2 cm of the frontal lobe, represents the state of oxygenated hemoglobin in the microvasculature, and approximates CBF [52]. Therefore, continually integrating fluctuations between MAP and rSO_2_, a Pearson’s product-moment correlation coefficient is generated. This correlation coefficient (COx) varies between −1 and +1. Positive COx values, where there is a positive and linear correlation between MAP and rSO_2_, indicate dysfunctional autoregulation [53]. Near-zero and negative COx values indicate intact autoregulation (i.e., rSO_2_ remains relatively constant despite varying MAP). The optimal MAP is identified as the MAP with the lowest value of COx, as shown in Fig. 3. Lee et al. demonstrated that COx identified the lower limit of autoregulation in a swine model of pediatric HIBI [53]. Recently, Ameloot et al. retrospectively calculated COx using MAP and rSO_2_ to indicate that autoregulation was intact in 33 of 51 subjects with HIBI. Thereafter, Pham et al. showed that COx was significantly higher in nonsurvivors of HIBI than in survivors [54]. Although higher COx was associated with nonsurvivors, there was no association between rSO_2_ and mortality. Recently, our research team demonstrated feasibility of monitoring COx in real time and identification of optimal MAP prospectively in 20 patients after CA [55]. Subjects spent approximately 50% of time outside a ±5 mmHg range from the optimal MAP, and, importantly, the optimal MAP was consistently identified in 19 of 20 subjects. The concept of individualized perfusion pressures is emerging as an attractive therapeutic target and improved clinical outcome is associated if actual MAP is maintained within proximity of the identified optimal MAP. It is imperative to recognize the downsides of targeting significantly right-shifted optimal MAP, particularly in patients with compromised left ventricular function after CA. Increasing afterload on a decompensated left ventricle can dramatically reduce stroke volume and cardiac output, placing the injured brain at increased risk of ischemia. Therefore, increased MAP targets in HIBI should be weighed against concurrent myocardial function. Considerable work remains to further delineate if individualized perfusion targets decrease brain hypoxia and secondary injury and are associated with improved neurologic outcome.

### Temperature

Targeted temperature management has historically been the focus of considerable HIBI research. It is a mainstay in the management of HIBI by mitigating secondary injury after CA [56]. At the cellular level, the beneficial effects of hypothermia are well documented. Cerebral metabolism is reduced by 5% to 10% per 1 °C decrease in core body temperature. In addition, global carbon dioxide production and oxygen consumption are decreased proportionally to reductions in core body temperature [57]. By decreasing cerebral metabolism, hypothermia avoids excessive intracellular anaerobic metabolism, which leads to increased lactate production. Hypothermia also improves cerebral glucose use and allows available cellular energy stores to be used for necessary cellular functions in keeping with neuronal survival [56]. Additional benefits of hypothermia include prevention of apoptosis by decreasing proapoptotic mediators such as p53, tumor necrosis factor α, and caspase enzymes while increasing expression of antiapoptotic proteins such as Bcl-2 [56, 57]. Hypothermia also prevents mitochondrial dysfunction, a key pathway involved in the promotion of apoptosis by release of cytochrome c oxidase into the cellular cytoplasm [56]. Finally, hypothermia decreases inflammatory mediators such as the interleukin-1 family of cytokines [58] as well as chemotaxis of leukocytes into cerebral interstitial tissue [56], reduces excitotoxic neurotransmitter release (glutamate and glycine) [57], and decreases free radical production after HIBI [57]. Sustained hypothermia also has detrimental physiologic effects pertaining to immune suppression, hemoconcentration, coagulopathy, arrhythmias, electrolyte disturbances, and hemodynamic instability, which must be weighed against the possible benefits [56]. Furthermore, unintentional hypothermia can occur after CA, indicating possible severe damage to the key centers of thermoregulation, including the hypothalamus [56].

Hyperthermia is associated with numerous pathophysiologic sequelae that are potentially harmful after HIBI. Specifically, hyperthermia may increase blood-brain barrier permeability, leading to worsening cerebral edema, ICP,, and cerebral ischemia. Furthermore, hyperthermia increases glutamate production, which in turn causes intracellular Ca^2+^ influx, leading to neuronal cell death, seizures, and further secondary injury [3]. Increased cerebral metabolism, hyperemic blood flow, and increased ICP are additional downstream consequences of uncontrolled hyperthermia in HIBI [3]. Recently, we showed that hyperthermia is associated with dysfunctional autoregulation in patients with HIBI [55].

Clinical studies have established a firm link between hypothermia and improved outcome after CA. In 2002, two randomized controlled trials demonstrated marked improvement in clinical outcomes in patients with CA after ventricular fibrillation or ventricular tachycardia who were treated with hypothermia compared with standard of care [6, 59]. A persistent criticism of both studies was that the standard-of-care groups maintained core body temperatures >37 °C, thereby exposing patients to the harmful effects of hyperthermia. This prompted a third recent randomized controlled trial comparing core body temperature control of 36 °C (normothermia) versus 33 °C (hypothermia) after CA [7]. This pragmatic trial included patients with HIBI with all initial cardiac rhythms and ultimately did not demonstrate an appreciable benefit of hypothermia versus normothermia [7]. Importantly, it must be stated that the maintenance of normothermia at 36 °C after CA requires active cooling. The negative effects of sustained hyperthermia and adverse outcomes after CA are well established [60, 61], thereby reinforcing the importance of aggressive core body temperature control in patients following CA. It is possible that individualized temperature targets exist within patients with HIBI, and the inability of current studies to concurrently monitor cerebral metabolism, ICP, and biomarkers of neuron degeneration has limited our ability to make these patient-specific distinctions.

### Normobaric hyperoxia

The dissolved portion of oxygen in plasma is a minor contributor to overall oxygen content. However, in disease states, this portion may have a pivotal role in ensuring adequate hemoglobin saturation for CDO_2_ and overcome diffusion barriers to restore normal cellular metabolism. Augmenting arterial oxygen content is touted as a crucial modifiable factor in optimizing CDO_2_ after HIBI, with normobaric hyperoxia being suggested to achieve this goal.

Upon ROSC, reperfusion injury occurs as a result of oxygen free radical production, which leads to intracellular oxidation [62]. Examples include superoxide (O_2_^−^), hydrogen peroxide (H_2_O_2_), hydroxyl anion (OH^−^), and nitrite (NO_2_^−^). Endogenous antioxidants balance the generation of free radicals and stabilize cellular function. Inadvertent normobaric hyperoxia in HIBI may tip this balance in favor of free radical production, cellular oxidation, and neuronal death [62]. Although a systematic review of animal studies of HIBI suggested that increased neuron dysfunction occurs after normobaric hyperoxia, there was significant between-study heterogeneity with respect to ventilation strategies, timing and dose of normobaric hyperoxia, concomitant use of hypothermia, and the chosen primary outcomes [63]. There are also several reported adverse effects associated with normobaric hyperoxia, including increased vascular resistance (cerebral, myocardial, and systemic), decreased CBF, seizures, and increased release neuronal degeneration biomarkers such as neuron-specific enolase [57, 62, 64, 65].

Researchers in several studies have evaluated normobaric hyperoxia in HIBI, with conflicting results. Kuisma et al. conducted a randomized study of patients who were given 21% or 100% inspired oxygen after ROSC [66]. The group that received 21% inspired oxygen exhibited lower serum levels of neuron-specific enolase than the normobaric hyperoxia group that did not undergo concomitant hypothermia. Kilgannon et al. interrogated the Project IMPACT database with more than 400,000 patients [67]. They included patients with nontraumatic CA and cardiopulmonary resuscitation within 24 h prior to intensive care admission. Their objective was to examine the association between hyperoxia and mortality. Compared with the subjects in the normoxia group, subjects with normobaric hyperoxia (partial pressure of arterial oxygen [PaO_2_] >300 mmHg) had higher associated in-hospital mortality (OR 1.8, 95% CI 1.5–2.2). Compared with normoxia, hypoxia (PaO_2_ < 60 mmHg) was also associated with increased in-hospital mortality (OR 1.3, 95% CI 1.1–1.5). Spindelboeck et al. studied normobaric hyperoxia and hypoxemia during CA and found that both were associated with increased mortality [68], suggesting that the deleterious effects of normobaric hyperoxia may occur in early stages of HIBI. Finally, Bellomo et al. conducted a retrospective analysis of patients with CA and demonstrated that normobaric hyperoxia and hypoxemia were associated with increased mortality; however, after adjustment, this relationship was no longer significant [69]. Importantly, significant limitations in methodology should be noted, particularly the retrospective nature of these studies, the limitation of using mortality as a primary outcome in a brain injury population, and the fact that the definition of normobaric hyperoxia with a single PaO_2_ > 300 mmHg does not capture the true biological exposure of patients to normobaric hyperoxia after CA. Furthermore, hypothermia was not routinely used in the aforementioned studies.

Additional retrospective analyses investigating the use of normobaric hyperoxia with concomitant hypothermia have addressed this shortcoming. Janz et al. demonstrated an association between adverse neurologic outcome and normobaric hyperoxia administration [70]. These results are contrasted by those reported by Ihle et al. and Lee et al., who failed to show an association between normobaric hyperoxia and adverse neurologic outcome with concomitant hypothermia [71, 72]. Thereafter, a prospective study revealed an association between favorable neurologic outcome and higher mean PaO_2_ [73]. Thus, concomitant hypothermia may play a role in modifying the deleterious effects of normobaric hyperoxia in HIBI.

## Conclusions

HIBI pathophysiology is complex, with a significant contribution attributable to secondary injury. Researchers have investigated the effects of interventions aimed at preventing secondary injury, most notably hypothermia. Future targets of research include individualized perfusion targets, normobaric hyperoxia, transfusion triggers, and PaCO_2_ goals.

## Abbreviations

ATP: Adenosine triphosphate
CA: Cardiac arrest
CBF: Cerebral blood flow
CDO_2_: Cerebral oxygen delivery
CMRO_2_: Cerebral metabolic rate of oxygen uptake
Cox: Correlation coefficient
CPP: Cerebral perfusion pressure
HIBI: Hypoxic ischemic brain injury
ICP: Intracranial pressure
MAP: Mean arterial pressure
PaCO_2_: Partial pressure of arterial carbon dioxide
PaO_2_: Partial pressure of arterial oxygen
RBC: Red blood cells
ROSC: Return of spontaneous circulation
rSO_2_: Regional saturation of oxygen
WBC: White blood cells
